# Health disparities and clinical trial recruitment: Is there a duty to tweet?

**DOI:** 10.1371/journal.pbio.2002040

**Published:** 2017-03-01

**Authors:** Arthur Caplan, Phoebe Friesen

**Affiliations:** Division of Medical Ethics, New York University School of Medicine, New York, New York, United States of America

## Abstract

While it is well known that the homogeneity of clinical trial participants often threatens the goal of attaining generalizable knowledge, researchers often cite issues with recruitment, including a lack of interest from participants, shortages of resources, or difficulty accessing particular populations, to explain the lack of diversity within sampling. It is proposed that social media might provide an opportunity to overcome these obstacles through affordable, targeted recruitment advertisements or messages. Recruiters are warned, however, to be cautious using these means, since risks related to privacy and transparency can take on a new hue.

It looks like there might be.

The homogeneity of clinical trial participants has long been recognized as both an ethical and scientific issue. Trials conducted in the United States that aim to better understand our health and how to improve it primarily involve middle- and upper-class, younger, white male participants, leading to skewed data. Information that can benefit huge numbers of people is not obtained, harming both those involved in the research process who lack a broad baseline for comparisons and those left out. Relying on limited samples of participants within biomedical research leads to the development of a body of clinical knowledge that may not be generalizable but that is often treated as such. This means that overall needs are less well understood, the ability to identify and compare variations is nonexistent, and available interventions are limited in scope. Including diverse populations in clinical research may lead to better, more robust data, greater equality, and, eventually, fewer disparities in health outcomes.

Despite the Revitalization Act of 1993, which required that clinical trials funded by the National Institutes of Health include women and minorities as participants, significant progress has not been made [1]. An analysis of 86 federally funded randomized control trials published in 2009 found that 75% didn’t offer any reporting of outcomes by sex, while 64% offered no analysis of the data in terms of race or ethnicity. Only three of these publications acknowledged that a lack of diversity may impact the generalizability of the reported results [2]. Many drugs have been withdrawn from the market only after the distinct health risks for women became apparent, results that did not surface in clinical trials primarily composed of men [3]. The underexplored connection between racial and ethnic disparities and illness can readily be seen in the area of cardiovascular health. In 31 North American cardiovascular cohort studies analyzed by Ranganathan et al., 18 restricted sampling to only whites or included no report or analysis of race or ethnicity [4], despite evidence suggesting that cardiovascular health varies significantly between different ethnic groups [5].

Reasons given for the exclusion of women and ethnic and racial minorities from research include a lack of interest from potential participants, physician bias, difficulties accessing these populations, and a lack of resources [4, 6–8]. However, there is growing evidence that suggests that a lack of interest from participants is unlikely to be a significant contributor to the exclusion of racial and ethnic minorities. When asked, blacks and Hispanics within the US are just as likely as whites to say they are willing to participate in clinical trials [9–11], a surprising finding for many, given the history of the mistreatment of minorities within research settings in the US (e.g., The Tuskegee Syphilis Experiment) and the lower levels of trust in physicians reported in minority populations [9].

Additionally, the underrepresentation of minorities in clinical trials is not straightforward. While it is true that minorities in the US make up a small proportion of the samples recruited for Phase II and III clinical trials, those which have the most potential to benefit participants, minorities are in fact overrepresented in Phase I trials, which involve the greatest risks and the lowest likelihood of benefit. These early trials often do not provide treatment or compensation to those injured as a result of participation [12, 13]. This suggests that not only is there a duty to include more minorities in research (within Phase II and III trials), but there may also be an obligation to broaden the number of non-minorities participating in Phase I trials.

The solution to some of these recruitment issues is likely right in front of you. If you’re like most Americans, you’ve already stared at it today [14]—Facebook might be the answer.

Social media sites have enormous potential for balancing out unfair sampling within clinical trials. With 86% of Americans online [14] and four-fifths of them using the internet to look for health information [15], their reach is vast, offering researchers an opportunity to access potential participants with unprecedented precision. As some have noted, “social media may provide an infrastructure that allows investigators to interact with the public in new ways, including stimulating interest in new clinical trials with **targeted** messages to connect patients, caregivers, and families with potential trial enrollment websites” (our emphasis) [16].

The potential for targeted messages and advertisements grants those tasked with recruitment for clinical trials an incredible amount of control over how recruitment materials are presented, to whom and when, making the task of recruiting a more diverse sample easier. Multiple advertisements can be designed with particular audiences in mind and shown exclusively to potential participants on the basis of age, location, language, education, relationship status, or occupation. Advertising to a particular group can be stopped once enough participants have been enrolled, and recruitment efforts can be directed towards populations that are under-enrolled.

While 79% of internet users have Facebook accounts, expanding recruitment efforts to Twitter and Instagram is likely to make diverse sampling even easier [14]. While 28% of black and Hispanic internet users have Twitter, only 20% of white people do. The rates are even higher on Instagram, with 47% of black internet users, 38% of Hispanic internet users, and only 21% of their white equivalents having an account [17]. Each of these sites offers the possibility of paid, targeted advertisements, as well as free accounts from which researchers can post recruitment notices, answer questions, and communicate with potential participants. Research teams have utilized both Facebook and Craigslist to recruit young female cancer survivors, despite this being a traditionally difficult population to recruit [18]. Several other researchers have also reported successful experiences recruiting and retaining diverse, hard-to-reach, and minority populations through social media [19–26].

Still, despite some success, little clinical trial recruitment is being done through social media. A recent article in the Journal of the American Medical Association Oncology reports that of 1,500 tweets containing the words “lung cancer” that were analyzed, nearly 18% of those related to clinical trials, but virtually none of these linked to recruitment sites (one tweet only) [16].

There are worries concerning the use of social media recruitment for clinical trials that may hinder its use. Issues of ensuring privacy may take on a different hue in online recruiting. Individuals who are posting publicly about their experiences of illness may not be aware that their words are available to the public, including researchers, and so may be alarmed if they see an advertisement or receive a message inviting them to participate in a clinical trial. Researchers need to take care to make their presence known in online spaces so that they are not perceived as invasive by those involved in intimate, supportive patient networks [27]. Surprising patients who believe their information was posted privately can also be avoided by posting recruitment notices to the Facebook page of a patient or advocacy group or by asking a community leader (who has many followers) to retweet, repost, or share a message or link [28]. Limits in terms of space (e.g., advertisements) and character counts (e.g., tweets) may also threaten researchers’ transparent disclosures, since initial recruitment materials will inevitably include only some information related to trial risks, benefits, and exclusions. Online recruiters must be especially careful to stay within the bounds of the Food and Drug Administration guidelines regarding clinical trial recruitment. Participants must never be induced by the promise of a cure or led to believe they are being given “free medical treatment.” They must always be fully informed of the chances that they will be given an experimental intervention and the chances that they will be given a placebo treatment [29].

Inequalities related to race and gender exist at every stage of clinical trials. Researchers have an obligation to remedy these inequalities, both for the sake of women and minorities who deserve to benefit from the research burdens they bear and in order to better contribute to generalizable knowledge. The potential to target particular populations through social media is a tool that can help researchers fulfill this obligation. Done in an ethically sensitive manner, social media can aid recruitment efforts in order to involve a truly representative sample of the population they aim to investigate.
